# The Interplay of Host Lysosomes and Intracellular Pathogens

**DOI:** 10.3389/fcimb.2020.595502

**Published:** 2020-11-20

**Authors:** Kuldeep Sachdeva, Varadharajan Sundaramurthy

**Affiliations:** National Centre for Biological Sciences, Tata Institute of Fundamental Research, Bangalore, India

**Keywords:** lysosomes, *M. tuberculosis*, *Salmonella*, heterogeneity, transcription factor EB, lysosomal homeostasis

## Abstract

Lysosomes are an integral part of the intracellular defense system against microbes. Lysosomal homeostasis in the host is adaptable and responds to conditions such as infection or nutritional deprivation. Pathogens such as *Mycobacterium tuberculosis* (*Mtb*) and *Salmonella* avoid lysosomal targeting by actively manipulating the host vesicular trafficking and reside in a vacuole altered from the default lysosomal trafficking. In this review, the mechanisms by which the respective pathogen containing vacuoles (PCVs) intersect with lysosomal trafficking pathways and maintain their distinctness are discussed. Despite such active inhibition of lysosomal targeting, emerging literature shows that different pathogens or pathogen derived products exhibit a global influence on the host lysosomal system. Pathogen mediated lysosomal enrichment promotes the trafficking of a sub-set of pathogens to lysosomes, indicating heterogeneity in the host-pathogen encounter. This review integrates recent advancements on the global lysosomal alterations upon infections and the host protective role of the lysosomes against these pathogens. The review also briefly discusses the heterogeneity in the lysosomal targeting of these pathogens and the possible mechanisms and consequences.

## Biology of Lysosome

Lysosomes are membrane-bound acidic, catabolic subcellular organelles present in eukaryotic cells. Lysosomes were first discovered by Christian René de Duve in 1955 (Appelmans et al., 1955; De Duve et al., 1955). Subsequent work from de Duve clarified that even though lysosomes are in a continuum with endocytic and biosynthetic pathways, they are distinct organelles (De Duve, 1963; Kornfeld and Mellman, 1989). Lysosomes are the terminal station for trafficking from degradative endocytosis, autophagy and phagocytosis, and consequently receive diverse cargo from these pathways. Lysosomes contain many types of hydrolytic enzymes including lipases, proteases, glycosidases, nucleases and sulfatases. These enzymes enable lysosomes to digest complex and diverse cargos (Luzio et al., 2017; Münz, 2017; Naslavsky and Caplan, 2018; Inpanathan and Botelho, 2019) into their constituent building blocks (Baggiolini, 1985; Kornfeld and Mellman, 1989; Casciola-Rosen and Hubbard, 1991; Rohrer and Kornfeld, 2001; Kolter and Sandhoff, 2005; Xu and Ren, 2015), which are released into the cytoplasm and recycled *via* the anabolic pathways of the cell (Luzio et al., 2017; Sabatini, 2017; Lawrence and Zoncu, 2019). Lysosomal acid hydrolases, which are synthesized in the endoplasmic reticulum, are tagged with mannose-6-phosphate (M6P) in the Golgi-network and then traffic to lysosomes *via* the endocytic system (Chao et al., 1990; Pohlmann et al., 1995; Saftig and Klumperman, 2009; Pohl and Hasilik, 2015; Luzio et al., 2017; Bhamidimarri et al., 2018). M6P receptor independent delivery of lysosomal proteins through sortilin and lysosomal integral membrane proteins (LIMPs) has also been reported (Glickman and Kornfeld, 1993; Storch and Braulke, 2005; Reczek et al., 2007; Braulke and Bonifacino, 2009; Saftig and Klumperman, 2009; Blanz et al., 2015; Markmann et al., 2015). Thus, the biogenesis of lysosomes requires the integration of endocytic and biosynthetic pathways (Kornfeld and Mellman, 1989; Saftig and Klumperman, 2009).

## Lysosomal Adaptation and Biogenesis Upon Signals

**Figure 1:**
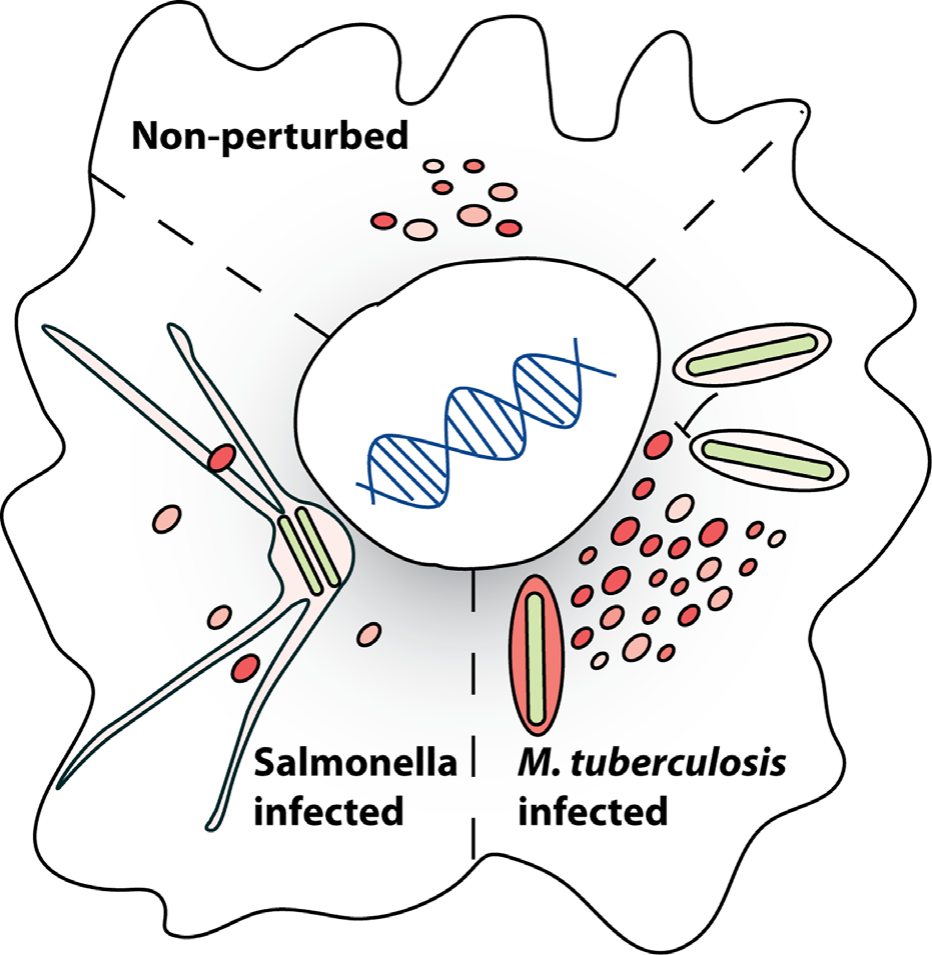
Infection induced alterations in lysosomal homeostasis. Lysosomal homeostasis in a eukaryotic cell is altered by different pathogens. Examples of how the lysosomes are altered during infection with *M. tuberculosis* (Mtb) and Salmonella are shown, with Mtb infection inducing lysosomal biogenesis and Salmonella infection resulting in decreased lysosomal levels. Mechanisms and consequences of these modifications are discussed in the review. Lysosomal heterogeneity in a cell under non-perturbed conditions is depicted by the slightly different sizes and colors of the lysosomes.

Long considered as the “garbage bin” of the cell, the role of lysosomes has undergone tremendous revisions over the last few years. Lysosomes play an important role in maintaining homeostasis of several cellular processes including cellular clearance, metabolism, plasma membrane repair, pathogen defense, bone remodeling and act as signaling platform (Baron et al., 1985; Reddy et al., 2001; McNeil, 2002; Kurz et al., 2011; Settembre and Ballabio, 2011; Lacombe et al., 2013; Xu and Ren, 2015; Diez-Roux and Ballabio, 2016; Erkhembaatar et al., 2017; Palmieri et al., 2017; Thelen and Zoncu, 2017; Tsukuba et al., 2017; Corrotte and Castro-Gomes, 2019; Lawrence and Zoncu, 2019). An adaptive and dynamic lysosomal response upon ever-changing cellular environment is critical for the maintenance of cellular homeostasis, one such response being the biogenesis of lysosomes itself (Settembre et al., 2013; Inpanathan and Botelho, 2019). A major environmental trigger for lysosomal biogenesis is infections with intracellular pathogens such as *M. tuberculosis* and Salmonella (**Figure 1**).

Endo-lysosomal proteins such as Rab7 and Lamp proteins play crucial roles in maintenance of lysosomal homeostasis. Rab7 GTPase plays a key role in trafficking of lysosomal enzymes from endosomes to lysosomes, as expression of dominant negative Rab7 impairs lysosomal delivery of acid hydrolases (Press et al., 1998), acidification and ultimately degradation of cargo in lysosomes (Bucci et al., 2000; Guerra and Bucci, 2016). Similarly, trafficking of a subset of lysosomal enzymes and recycling of M6P receptors is altered upon Lamp2 deficiency (Eskelinen et al., 2002), showing its essential nature.

**Figure 2:**
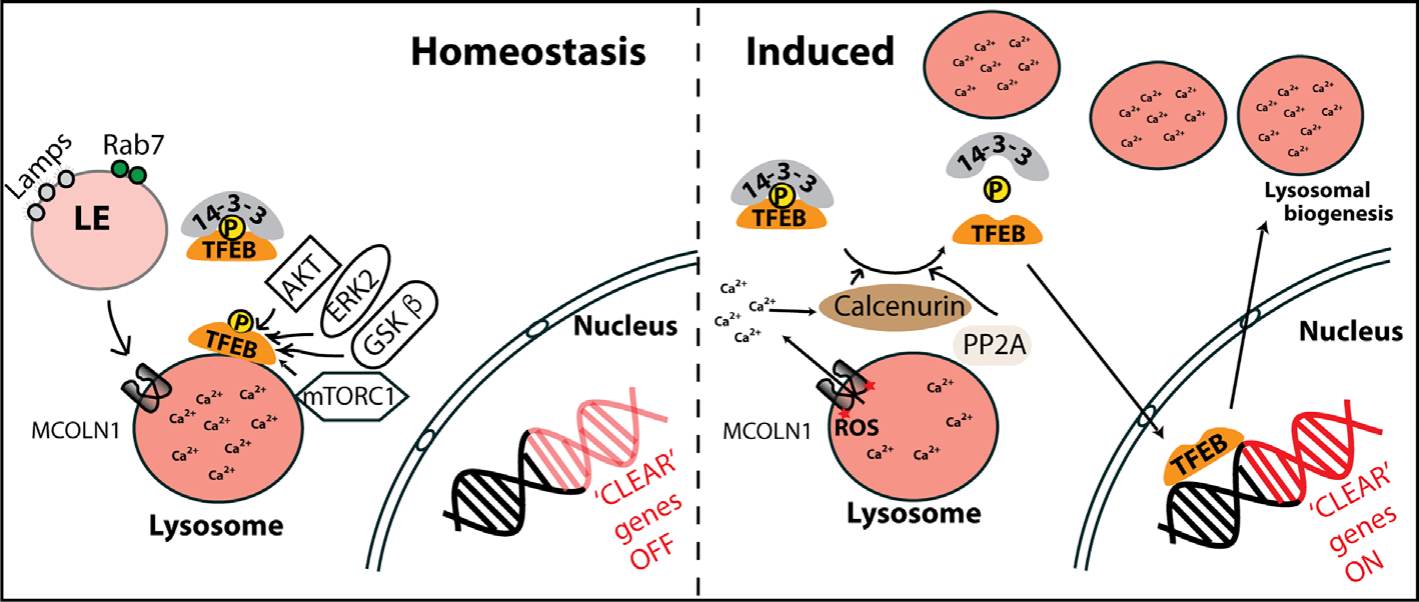
Regulation of cellular lysosomal homeostasis by transcription factor EB (TFEB). In homeostatic conditions, late endosomes (LE) containing lysosomal associated membrane proteins (Lamps) and Rab7 fuse with lysosomes. Multiple kinases including mTORC1, AKT, ERK2, GSKβ phosphorylate TFEB, retaining it on the lysosomal surface or sequestered to 14-3-3 chaperons in the cytoplasm. In conditions of lysosomal induction such as starvation, mTORC1 is inactivated and ROS activated MCOLN1 channel releases Ca^2+^ from lysosomal lumen to cytoplasm. The release of lysosomal Ca^2+^ locally activates calcineurin, which dephosphorylates TFEB. Protein phosphatase 2A (PP2A) is another reported TFEB phosphatase. Dephosphorylated TFEB translocates to the nucleus where it induces transcription of genes containing CLEAR elements, that include several lysosomal genes, eventually resulting in enhanced lysosomal biogenesis.

The mechanisms regulating cellular lysosomal biogenesis are becoming clear in the recent years. Transcription factor EB (TFEB), a basic helix-loop-helix-leucine zipper transcription factor of the microphthalmia family, regulates transcription of the lysosomal genes and subsequently lysosomal biogenesis in cell. Multiple kinases including mTORC1, ERK2, AKT, GSKβ and PKCβ phosphorylate TFEB at different residues and regulate its subcellular localisation (Settembre et al., 2011; Parr et al., 2012; Ferron et al., 2013; Li et al., 2016; Palmieri et al., 2017; Puertollano et al., 2018). In nutrient rich conditions, activated mTORC1 on the lysosomal membrane phosphorylates TFEB at Ser142 and Ser211, which promotes the binding of TFEB with the 14-3-3 cytosolic chaperon and favors its cytoplasm retention. Conversely, mTORC1 inactivation upon starvation leads to nuclear translocation of TFEB (**Figure 2**) (Settembre and Ballabio, 2011; Roczniak-Ferguson et al., 2012; Settembre et al., 2012; Puertollano et al., 2018). Dephosphorylation of TFEB by calcium-activated calcineurin and protein phosphatase 2A also induces nuclear translocation of TFEB (Medina et al., 2015; Zhang et al., 2016; Martina and Puertollano, 2018; Najibi et al., 2019). In the nucleus, TFEB binds to Coordinated Lysosomal Expression and Regulation (CLEAR) element in promoter region of the lysosomal genes and subsequently induces their transcription (Sardiello and Ballabio, 2009). Thus, signals from different cascades integrate at TFEB to regulate the lysosomal biogenesis and homeostasis in cells (**Figure 2**). Emerging evidence indicate that infection by pathogens influence these lysosomal signaling pathways in the cells (Campbell et al., 2015; Najibi et al., 2019; Sachdeva et al., 2020a). In the following sections, we will explore the intracellular trafficking of two specific pathogens and their intersection with lysosomal pathways.

## Dynamics of PCV and Host Trafficking Alterations

In the host cell, several innate immune elements such as reactive oxygen and nitrogen species (ROS, RNS), lysosomal acid hydrolases, xenophagy, inflammasome, septin caging, and proteasomal degradation are employed to neuter the pathogens (Radtke and O’Riordan, 2006; Deretic and Levine, 2009; Kaufmann and Dorhoi, 2016). But most pathogens manipulate these host mechanisms and generate an intracellular milieu conducive for their survival. One common strategy employed by many pathogens is to avoid trafficking to lysosomes (Méresse et al., 1999b; Duclos and Desjardins, 2000; Alix et al., 2011; Tang, 2015; Martinez et al., 2018). Several pathogens secrete effectors that target key host factors such as Rab GTPases (Deretic et al., 1997; Via et al., 1997; Fratti et al., 2001; Vergne et al., 2003b; Smith et al., 2007; Seto et al., 2011; Sherwood and Roy, 2013; Pradhan et al., 2018), cytoskeleton and motor protein (Dramsi and Cossart, 1998; Bearer and Satpute-Krishnan, 2002; Rottner et al., 2005; Henry et al., 2006b; Haglund and Welch, 2011; Shimono et al., 2016; Anes, 2017; Zhou et al., 2018) and block the fusion with lysosomes (Via et al., 1997; Russell, 2001; Pieters, 2008; Pradhan et al., 2018), instead residing in a modified phagosome, often called the pathogen containing vacuole (PCV) (Haas, 2007).

**Figure 3:**
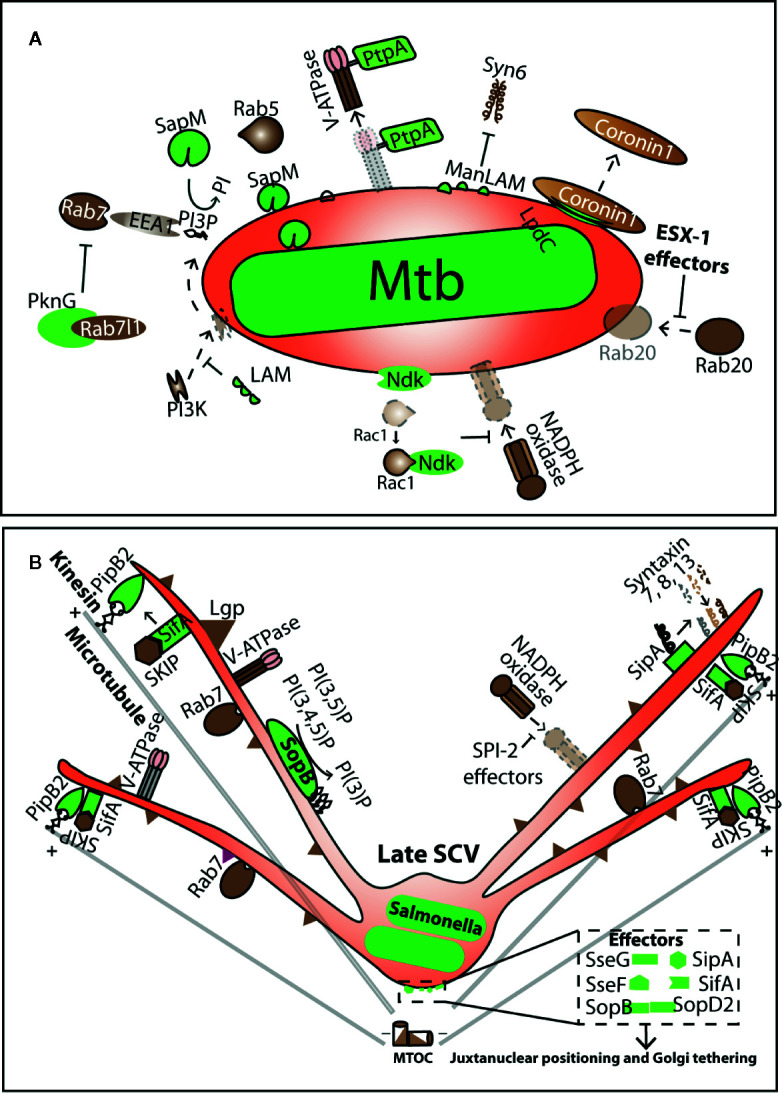
Manipulation of lysosomal trafficking by Pathogen containing vacuoles. **(A, B)** summarizes some of the known molecular events in the interface of Mtb containing vacuole (MCV) and Salmonella containing vacuole (SCV), respectively. Pathogen effectors (shown in green) target different host factors (brown) and influence their recruitment or release on the respective PCV’s. Arrows indicate the nature of interactions. Dotted arrows show the processes that occur on a normal phagosome but are manipulated on the PCV.

**Figure 4:**
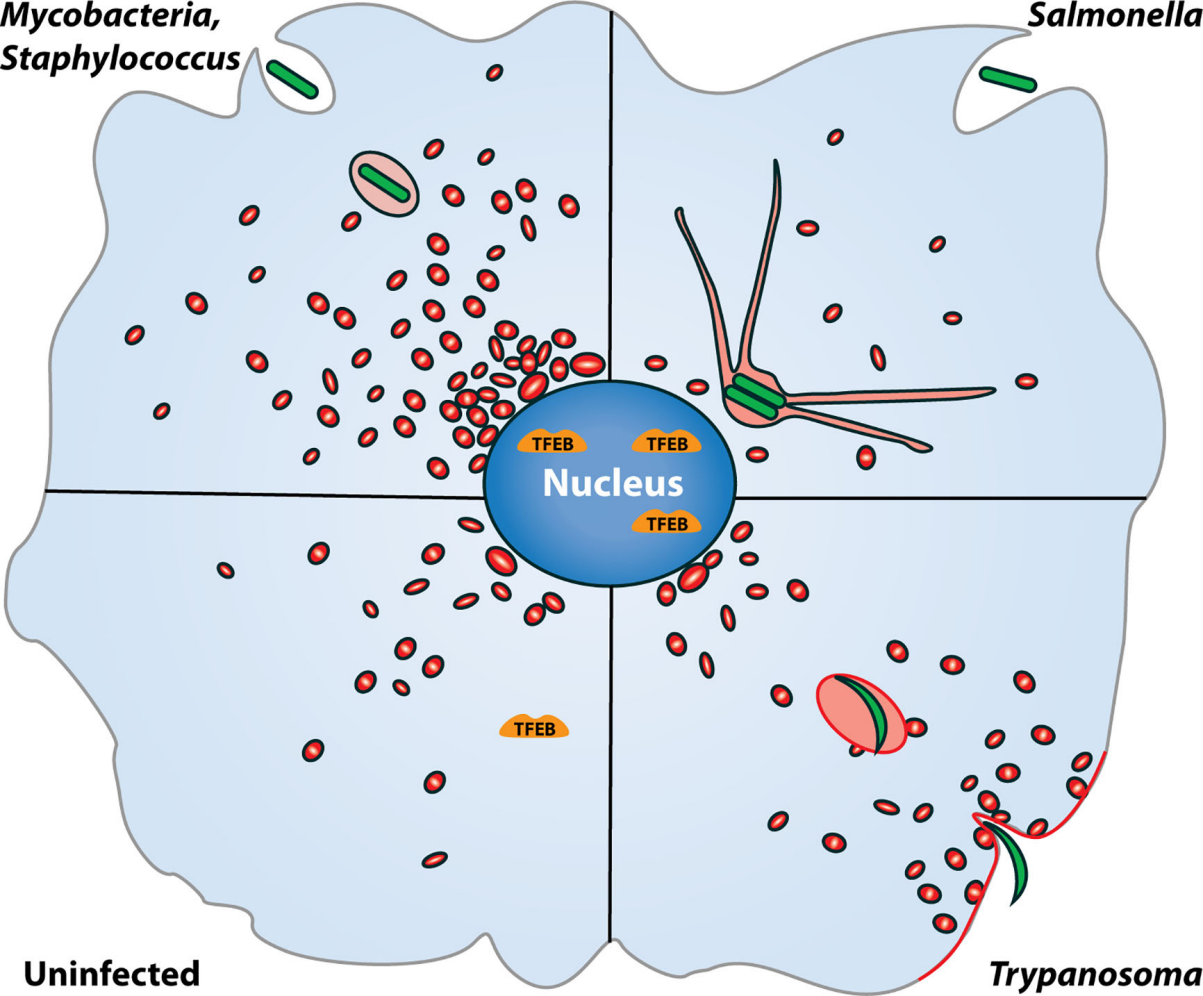
Alteration in the host lysosomal system upon intracellular infections. The host lysosomal alterations in four different pathogenic infections are shown. Lysosomes are shown in red. *Mtb* and *Staphylococcus aureus* infection increases the host cellular lysosomes levels compared to uninfected condition, whereas *Salmonella* infections leads to formation of late endosome/lysosome derived filamentous structures. In *Trypanosoma* infection, lysosomes move toward cell periphery at the site of pathogen entry and fuse with the plasma membrane to facilitate pathogen entry in the host cell. In these infectious condition, i.e *Mtb*, *Salmonella*, *Staphylococcus* and *Trypanosoma*, transcription factor EB (TFEB) is translocated to nucleus as compared to the uninfected state.

**Figure 5:**
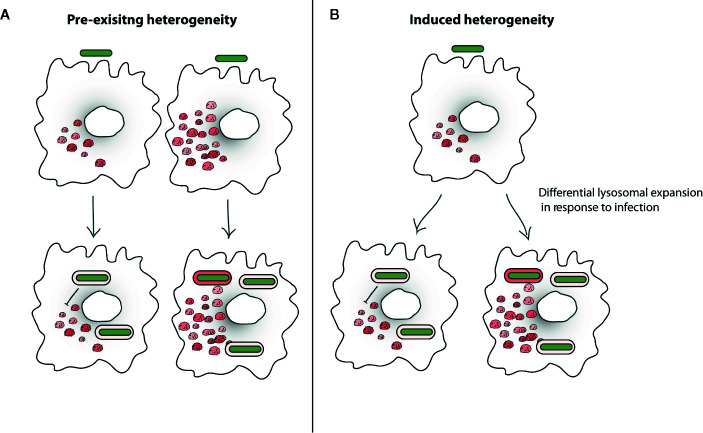
Lysosomal heterogeneity influencing intracellular *Mycobacterium tuberculosis* (Mtb) trafficking. **(A)** Heterogeneity in lysosomal targeting of pathogens in the host cells could be driven by pre-existing heterogeneity of host cells where some cells intrinsically have more lysosomes and are more efficient in trafficking *Mtb* (green) to lysosomes (red), or it could also be driven by pathogen diversity, where some pathogens cells are less capable of resisting lysosomal delivery. **(B)** Other model proposes the idea of induced heterogeneity where post-infection the heterogeneity is induced in the host and pathogen e.g. lysosomal levels and lysosomal delivery of *Mtb* in cells becomes heterogeneous. Existing literature suggests that these events are not mutually exclusive and pre-existing heterogeneity in the pathogen cells can induce diversity in the host cell response. Similarly, heterogeneity of the host cells can generate diversity in the pathogens upon infection.

*Mycobacteria* and *Salmonella* are two well-studied pathogens that intersect with and manipulate the endo-lysosomal system of the host for their survival. Intracellular trafficking alteration of the PCV and escape from lysosomal delivery is well studied in both these pathogens (Méresse et al., 1999b; Duclos and Desjardins, 2000; Hashim et al., 2000; Gorvel and Méresse, 2001; Fratti et al., 2003; Vergne et al., 2003b; Chua et al., 2004; Smith et al., 2005; Deghmane et al., 2007; Smith et al., 2007; Alix et al., 2011; Vergne et al., 2014; Tang, 2015; Martinez et al., 2018). In this review, we will focus on the interaction of these pathogens with the functioning of the host lysosomes (**Figure 1**). We will discuss the molecular mechanisms by which the two pathogens intersect with lysosomal trafficking to establish and sustain their respective PCV’s (**Figure 3**). While these mechanisms are well known, and other reviews have documented them extensively (Russell, 2001; Holden, 2002; Pieters, 2008; Steele-Mortimer, 2008; Lahiri et al., 2010; Queval et al., 2017; Upadhyay et al., 2018) less is known about the impact of these infections on the lysosomal homeostasis of the infected cell. We will discuss emerging evidence from literature on the impact of these intracellular pathogens on the lysosomal system globally in the infected cells, beyond the confines of the PCV’s and explore their consequences (**Figure 4**). Finally, we will briefly explore the emerging role of heterogeneity in shaping the outcomes of host-pathogen encounters, and examine the role of lysosomes in this context (**Figure 5**).

## *Mycobacteria* Containing Vacuole Trafficking and Maturation

While inert particles such as beads undergo the process of phagosomal maturation and eventually fuse with the lysosomes, *Mtb* employs several virulence factors such as PknG, PtpA, SapM, LpdC, ManLAM, Ndk, ESAT6, CFP10 (**Figure 3A**) (Via et al., 1997; Fratti et al., 2001; Fratti et al., 2003; Vergne et al., 2003b; Chua et al., 2004; Vergne et al., 2004; Vergne et al., 2005; Deghmane et al., 2007; MacGurn and Cox, 2007; Bach et al., 2008; Wong et al., 2011; Simeone et al., 2012; Mehra et al., 2013; Shukla et al., 2014; Pradhan et al., 2018) that impede its vacuolar maturation and fusion with the lysosomes (Armstrong and Hart, 1971; Russell, 2001; Pieters, 2008; Cambier et al., 2014; Awuh and Flo, 2016). These effectors modulate several host factors such as PI3Kinase (Fratti et al., 2003; Vergne et al., 2003a; Chua and Deretic, 2004; Zulauf et al., 2018), V-ATPase (Bach et al., 2008), EEA1, Rab7, Lamp2 (Via et al., 1997; Fratti et al., 2001; Seto et al., 2011; Hu et al., 2015; Pradhan et al., 2018), coronin-1 (Ferrari et al., 1999; Deghmane et al., 2007; Jayachandran et al., 2007) and SNAREs (Fratti et al., 2002; Fratti et al., 2003; Vergne et al., 2004) on *Mycobacteria* containing vacuole (MCV) (**Figure 3A**) to arrest maturation of the vacuole (**Figure 2**). Despite the arrested state, MCVs remain fusogenic to early and recycling endosomes, and in fact, show higher fusion with early endosomes (Vergne et al., 2004; Halaas et al., 2010). These reports convincingly show that *Mtb* extensively modulates its vacuole to attain effective maturation blockage ensuring that the majority of the bacteria do not fuse with lysosomes in cultured macrophages *in vitro*.

## *Salmonella* Containing Vacuole Trafficking and Maturation

*Salmonella* is another fascinating pathogen whose intracellular lifestyle intersects extensively with the endo-lysosomal system of host cells. *Salmonella* can infect non-phagocytic cells by secreting effectors in the host (Brumell et al., 1999; Fu and Galán, 1999; Zhou et al., 1999; Galan and Zhou, 2000). In non-phagocytic cells, *Salmonella* containing vacuoles (SCVs) acquire early and late endosomal markers e.g. lysosomal membrane glycoproteins, V-ATPases and acquire NADPH oxidase (Vazquez-Torres et al., 2000; Gallois et al., 2001) but do not fuse with lysosomes (Garcia-del Portillo and Finlay, 1995; Méresse et al., 1999a; Uchiya et al., 1999; Duclos and Desjardins, 2000; Gorvel and Méresse, 2001) (**Figure 3B**). The limited lysosomal hydrolytic activity in SCV could also be because of the rapid dilution of these enzymes in larger area of SCV (Krieger et al., 2014; Liss and Hensel, 2015; Knuff and Finlay, 2017). *Salmonella* effectors such as PipB2, SifA, SipA, SopB, SopD2, SseJ, SseF, and SseG etc., target several host factors e.g. cytoskeleton, motor proteins, ESCRT machinery (Dukes et al., 2006), SNAREs (Singh et al., 2018), Rab GTPases (Smith et al., 2007) and phosphoinositide levels (Dukes et al., 2006) to maintain juxta-nuclear positioning and the integrity of the SCV (Beuzón et al., 2000; Salcedo and Holden, 2003; Guignot et al., 2004; Kuhle et al., 2004; Boucrot et al., 2005; Abrahams and Hensel, 2006; Abrahams et al., 2006; Henry et al., 2006a; Brawn et al., 2007; Ramsden et al., 2007; Steele-Mortimer, 2008; Wasylnka et al., 2008; Lau et al., 2019), *Salmonella* induced filaments (Sifs) formation (Stein et al., 1996; Waterman and Holden, 2003; Boucrot et al., 2005; Rajashekar et al., 2008; Szeto et al., 2009; Dumont et al., 2010; Leone and Méresse, 2011; Krieger et al., 2014; Rajashekar et al., 2014; Liss et al., 2017; Gao et al., 2018) and to inhibit fusion with lysosomes (Garcia-del Portillo and Finlay, 1995; Méresse et al., 1999a; Uchiya et al., 1999; Duclos and Desjardins, 2000; Gorvel and Méresse, 2001) (**Figure 3B**). Interestingly, SCVs are differentially modulated in epithelial cells and macrophages indicating potential cell type specific mechanisms (Reuter et al., 2020).

Altogether, these studies demonstrate that *Mtb* and *Salmonella* containing vacuoles are substantially transformed compared to bead containing phagosomes and these alterations have an astounding influence on the intracellular trafficking of these vacuoles along the lysosomal pathway.

While phagosome maturation arrest mediated by *Mtb* is well-studied in cultured macrophages *in vitro*, emerging evidence suggest that under *in vivo* infections, *Mycobacterium* could encounter acidic conditions and indeed be delivered to and adapt to lysosomal conditions (Levitte et al., 2016; Sundaramurthy et al., 2017). Indeed *Mtb* infected cells and tissues show higher lysosomal content *in vivo* (Sundaramurthy et al., 2017; Sachdeva et al., 2020a), and *Mtb* are delivered rapidly to lysosomes in a susceptible mouse model (Sundaramurthy et al., 2017). Lysosomal delivery however does not result in the elimination of *Mycobacteria*, rather a reduced growth rate (Levitte et al., 2016; Sundaramurthy et al., 2017). Using elegant photobleaching experiments in zebrafish infected with *M. marinum* (*Mm*), Ramakrishnan group showed that the bacteria residing in lysosomes have slower growth compared to the ones that are not fused to lysosomes (Levitte et al., 2016). These studies suggest that additional facets of *Mycobacterium* interaction with the lysosomal trafficking system could be manifest during *in vivo* infection, which warrants further investigations.

## Pathogens Affecting Host Lysosomal Homeostasis

While extensive work, as described above, has uncovered the mechanisms by which pathogens avoid lysosomal delivery, if and how these pathogens globally influence the host lysosomal homeostasis is not very-well understood. Documenting the global manipulations in the host cell by pathogens, beyond the confines of the PCV, is important to gain a complete appreciation of the influence of infection on the host, which further opens the possibilities to better design the counter-strategies against the pathogens. Recent work from several groups, including ours, have systematically addressed this question.

ESX-1 secretion system is known to play a role in impacting cellular lysosomes in different ways. *Mtb* and *M. marinum (Mm)* induces exocytosis of lysosomes upon infection in a ESX-1 dependent manner (Koo et al., 2008a). Esat-6, one of the well-studied substrates of the ESX-1 secretion system, has been recently shown to play a role in permeabilization of lysosomal membrane and release of mature cathepsin B into the cytosol, resulting in inflammasome activation and secretion of pro-inflammatory cytokines such as IL-1β (Amaral et al., 2018). Our recent work shows that *Mtb* infection alters the host lysosomal homeostasis and globally elevates the cellular lysosomal content of the infected macrophages under both *in vitro* and *in vivo* infections in mouse model. *Mtb* lipids, predominantly Sulfolipid-1, play a major role in lysosomal expansion. Correspondingly, a Sulfolipid-1 deficient *Mtb* mutant (*MtbΔpks2*) showed attenuated lysosomal rewiring. Sulfolipid-1 induces lysosomal biogenesis in macrophages in a mTORC1-TFEB dependent manner (Sachdeva et al., 2020a). Other bacterial components such as peptidoglycan from *Staphylococcus aureus* and lipopolysaccharide (LPS) induce lysosomal expansion using TFEB dependent as well as independent pathways. However, the role of peptidoglycan and LPS on lysosomes in an infection context remains to be validated. These studies show that bacterial membrane components have an influence on the signaling pathways regulating lysosomal homeostasis of the host cells (Hipolito et al., 2019; Najibi et al., 2019; Sachdeva et al., 2020a), and exert an effect on lysosomes globally in the infected cell, beyond the confines of the PCV’s.

*Salmonella* infection depletes acidic and catalytically active lysosomes in the host cells (Eswarappa et al., 2010; McGourty et al., 2012; Najibi et al., 2019). *Salmonella* effector PipB2 instigate tubulations of late endosome (LE)/lysosomes to form Sifs. Ectopic expression of PipB2 induces the dispersal of LE/lysosomes toward the cell periphery by increasing their net anterograde movement (Knodler and Steele-Mortimer, 2005). In addition, ectopic expression of SifA, SpiC, and SopD2 in mammalian cells also induces aggregation of LE/lysosomes (Brumell et al., 2001b; Boucrot et al., 2003; Brumell et al., 2003; Shotland et al., 2003). Few other studies have also reported remodeling of the endosomal system in *Salmonella* infection and propose that it facilitates the nutrient acquisition for the bacteria (Rajashekar et al., 2008; Liss and Hensel, 2015). Furthermore, *Salmonella*-effector SifA makes a stable complex with the host SKIP and Rab9 in infected cells and subverts the retrograde trafficking of mannose-6-phosphate receptors (MPRs). Subsequently, subverted MPR trafficking leads to misrouting of the lysosomal enzymes in the cell, which ultimately abolishes lysosomal catalytic activity (McGourty et al., 2012). Ectopic expression of SifA is enough to alter MPR trafficking and lysosomal function in HeLa cells (McGourty et al., 2012).

Other than *Mtb* and *Salmonella*, few other pathogens such as *Staphylococcus aureus, Plasmodium* and *Trypanosoma* alter lysosomal homeostasis in the host cells. Similar to *Mtb*, *Staphylococcus aureus* infection increases the levels of lysosomes in the host cells in a TFEB dependent manner (Visvikis et al., 2014; Najibi et al., 2016; Najibi et al., 2019). Infections by protozoan parasites such as *Plasmodium* and *Trypanosoma* alter lysosomal homeostasis by increasing lysosomes and inducing lysosomal exocytosis, which facilitates the entry of the pathogen in the host cells (Tardieux et al., 1992; Hissa et al., 2012; Vijayan et al., 2019). These reports suggest that different pathogens impose distinct alterations in the host lysosomal system beyond the confines of the pathogen containing vacuoles by influencing the signaling cascades regulating lysosomal homeostasis (**Figure 4**). Some of the factors from individual pathogens modulating lysosomal processes is summarized in **Table 1**, while much work remains to be done in this exciting and emerging area.

**Table 1:** Different pathogen and their effectors affecting host lysosomal homeostasis.

S. no	Pathogen	Effector	Effect on host lysosomes	References
1	*Mtb, Mm*	Esx-1 secretion system	Increases lysosomal exocytosis	(Koo et al., 2008b)
2	*Mtb*	ESAT-6	Increased lysosomal permeabilization	(Amaral et al., 2018)
3	*Mtb*	Sulfolipid-1	Increases lysosomal content of the host cell	(Sachdeva et al., 2020a)
4	*Mtb*	PIM6	Increases lysosomal content of the host cell	(Sachdeva et al., 2020a)
5	*Salmonella*	SPI-2 T3SS effector	Depletes acidic lysosomes in the host cell	(Eswarappa et al., 2010; Najibi et al., 2019)
6	*Salmonella*	SifA	Impairs MPR dependent trafficking of lysosomal enzymes and thereby lysosome function	(McGourty et al., 2012)
7	*Salmonella*	PipB2	Instigates tubulations of late endosome/lysosomes	(Knodler and Steele-Mortimer, 2005)
8	*Salmonella*	SifA, SpiC and SopD2	Induce aggregation of late endosome/lysosomes	(Brumell et al., 2001a; Boucrot et al., 2003; Shotland et al., 2003)
9	*Staphylococcus aureus*	Peptidoglycan	Increases lysosomal content of the host cell	(Najibi et al., 2019)
10	*Plasmodium yoelii*	Secretory effector(s)	Lysosomal exocytosis	(Vijayan et al., 2019)
11	*Trypanosoma cruzi (metacyclic)*	gp82 protein	Lysosome biogenesis/scattering	(Cortez et al., 2016)

## Role of the Global Lysosomal Alterations in Pathogenesis Mechanisms

The lysosomal system is modulated in response to the physiological state of the cells and signal integration at the level of TFEB to regulate the lysosomal homeostasis in the cell. Gray et al reported that phagocytosis of *Escherichia coli* induces lysosomal biogenesis in cells in a TFEB dependent manner and this lysosomal enrichment enhances the bactericidal properties of the host cell (Gray et al., 2016). Our recent work with *Mtb* mediated lysosomal enrichment shows that the increased lysosomal levels in *Mtb* infected cells have a host-protective role against pathogenic *Mtb* (Sachdeva et al., 2020a). *MtbΔpks2*, the SL-1 mutant *Mtb* strain, shows attenuated lysosomal rewiring and corresponds to a phenotype of enhanced intracellular *Mtb* survival (Sachdeva et al., 2020a). Lysosomal proteins such as Lamp1 and 2 are necessary for phagosomal fusion with lysosomes (Huynh et al., 2007). Importantly, they are regulated by TFEB (Palmieri et al., 2011; Vural et al., 2018). Hence, it is suggested that TFEB mediated lysosomal enrichment enhances the fusion of bacterial vacuoles with lysosomes and augments the lysosomal targeting of pathogens in the host cell (Vural et al., 2018). Interestingly phagosome maturation rate is accelerated during SL-1 mediated lysosomal expansion (Sachdeva et al., 2020a), suggesting that enhanced lysosomal function influences phagosome maturation and subsequently bacterial survival. In line with this, chemical inhibition of lysosomal acidification with bafilomycin and cathepsin D using pepstatin in primary macrophages increased the intracellular growth of *Mtb* (Welin et al., 2011). Similarly, blocking lysosomal enzyme β-hexosaminidase enhanced the intracellular survival of *M. marinum* suggesting lysosomal hydrolases mediated restriction mechanisms (Koo et al., 2008b). Thus, lysosomal enrichment in macrophages promotes the lysosomal targeting of a proportion of *Mtb* and consequently limits the intracellular replication of *Mtb* (Vural et al., 2018; Sachdeva et al., 2020a). Further lysosomal enrichment by chemical treatment of autophagy modulators nortriptyline and prochlorperazine edisylate (Sundaramurthy et al., 2013) or gefitinib (Sogi et al., 2017) in macrophages increased lysosomal targeting of *Mycobacteria* and suppressed the intracellular bacterial replication. Treatment with Interferon-gamma (IFN-*γ*), a cytokine of adaptive immunity, substantially decreases mycobacterial survival in the host (Flynn and Chan, 2001). Several findings suggest that the IFN-*γ* treatment substantially increases the anti-mycobacterial capacity of the host cells by enhancing lysosomal targeting of *Mycobacteria* (Schaible et al., 1998; Via et al., 1998). These studies suggest that pharmacologically increasing lysosomes influences the lysosomal delivery of *Mtb* and limits the growth of *Mtb* in cells, thus playing a protective role against *Mtb*. Thus either enrichment or depletion of lysosomal content has the respective opposite effect on intracellular Mtb survival, arguing for a reciprocal relationship between macrophage lysosomal content and intracellular Mtb survival.

The depletion of functional lysosomes upon *Salmonella* infection also facilitates the survival of the pathogen in the host (Eswarappa et al., 2010). Further, genetic or chemical modulation of the host lysosomes correspondingly affected the intracellular survival of *Salmonella* suggesting that the intracellular growth of *Salmonella* is affected by the lysosomes (McGourty et al., 2012). Several other studies have also reported that the chemical or genetic enrichment of lysosomes enhances bactericidal properties and bacterial clearance of other pathogens such as *Burkholderia cenocepacia*, methicillin-resistant *Staphylococcus aureus* and enteroinvasive *Escherichia coli* (Sogi et al., 2017; Vural et al., 2018; Liu et al., 2019). These studies show an emerging consensus with these limited examples of the global content of lysosomes in cells having a growth inhibitory effect on intracellular pathogens.

While these studies show the relevance of global alteration of lysosomes to the infection, our recent study also throws light on lysosomal heterogeneity influencing intracellular *Mtb* trafficking in a non-perturbed macrophage population, i.e a higher proportion of *Mtb* are delivered to lysosomes in cells with higher lysosomal content (Sachdeva et al., 2020a; Sachdeva et al., 2020b).

## Heterogeneity in Host-Pathogen Interaction

It is important to point out that lysosomal enrichment in cells, while restricting the intracellular growth of these pathogens, is not sufficient to completely eradicate the pathogen from the host cell. In fact, only a proportion of the intracellular *Mtb* population is targeted to lysosomes in response to the lysosomal enrichment in cells. Subcellular localization analysis in different studies shows that approximately 20-40% of *Mtb* are targeted to lysosomes (Welin et al., 2011; Sogi et al., 2017; Sachdeva et al., 2020a). When the lysosomal rewiring is attenuated, such as in *MtbΔpks2* mutant, even less *Mtb* are delivered to lysosomes (Sachdeva et al., 2020a). It would be interesting to see if the physiological state of the bacteria such as redox potential, metabolic activity or antibiotic sensitivity of this subpopulation is different from the rest of the *Mtb* population that is not delivered to lysosomes.

Recent studies reveal substantial heterogeneity in the pathogen and host cells at single cell level that impacts the outcome of host-pathogen encounters (Avraham et al., 2015; Avraham and Hung, 2016). Heterogeneity could be either pre-existing or infection induced. Avraham et al. showed that varying PhoPQ activity on LPS modification in a subset of *Salmonella* generates a heterogeneous population of the pathogens (Avraham et al., 2015). This heterogeneous *Salmonella* population upon infection further drives variable type 1 IFN response in the host cells, thus promoting diversity in the host cells (Avraham et al., 2015). This study is an elegant example of pathogen heterogeneity shaping the outcome of infection. Recent work from our laboratory reveals pre-existing endocytic heterogeneity in the macrophages that determine their susceptibility to infections (Sachdeva et al., 2020b). The study shows considerably high intrinsic heterogeneity in the endocytic capacity of individual cells in a population, which governs the subsequent phagocytic events, including intracellular infections and subcellular trafficking within macrophages (Sachdeva et al., 2020b). Interestingly, the cells with higher endocytosis also have high levels of lysosomes suggesting a co-regulation of the endosomal and lysosomal numbers (Sachdeva et al., 2020b). In line with this, a recent study indicates the transcription regulation of endocytic genes such as Rab5A and Rab7A by TFEB, a known regulator of lysosomal biogenesis (Nnah et al., 2018). However, the exact mechanisms determining the spread and extent of endo-lysosomal heterogeneity in cells are not known, although it is quite likely that fluctuations in the expression and subcellular localization of TFEB could play a role. Importantly, *Mtb* trafficking to lysosomes is different in cells with different lysosomal content, i.e a higher proportion of *Mtb* are delivered to lysosomes in cells with higher lysosomal content, even in the same non-perturbed macrophage cell population (Sachdeva et al., 2020b), an effect that averages out at a population level. Thus, the lysosomal content in *Mtb* infected cells can be a combination of pre-existing heterogeneity (Sachdeva et al., 2020b) and infection induced lysosomal biogenesis (Sachdeva et al., 2020a) (**Figure 5**). Importantly, differential subcellular trafficking of *Mtb* in these heterogeneous host cells could potentially generate diversity in the pathogen as well, since reports indicate that the physiological state of *Mtb* such as its redox potential is influenced depending on whether the bacterium is in lysosomes, or not (Bhaskar et al., 2014).

Similarly, a recent study shows another instance of pre-existing heterogeneity in the host primary human vascular endothelial cells (HUVEC), where susceptibility to *Listeria monocytogenes* infection is determined at the level of bacterial adhesion to cells (Rengarajan and Theriot, 2020). These studies demonstrate that heterogeneity, whether pre-existing or generated upon infection has a substantial impact on the pathogen-host encounters at multiple levels including uptake, sub-cellular trafficking and downstream responses such as gene expression changes. Further work and development of novel pathogen reporter strains are required to delineate between infection induced vs pre-existing heterogeneity in different contexts and gain quantitative insights into the consequences of encountering heterogeneous sub-cellular environments for the bacteria.

Overall, the role of heterogeneity in host-pathogen interactions at a single cell level –host or pathogen mediated and pre-existing or infection induced – determining population outcomes is emerging as a major theme in infection biology. How the heterogeneity in lysosome function and importantly composition influences, and is influenced by, infection events will be exciting area to explore in the future.

## Conclusion

In this review on intracellular pathogens and host lysosomal landscape, we show that pathogens such as *Mtb* and *Salmonella* impose global alteration on the host lysosomal system by manipulating the lysosomal signaling cascades in cells. These pathogens impose diverse kind of alterations in the host lysosomal landscape including enrichment, depletion and redistribution of the lysosomes. Importantly, lysosomes play a host protective role in the host-pathogen encounter and lysosomal enrichment in cells promotes delivery of pathogens such as *Mtb* and *Salmonella* to lysosomes, limiting the intracellular pathogen growth. Conversely, depletion of the host lysosomes promotes the intracellular survival of these pathogens. However, the lysosomal system does not completely eradicate pathogens such as *Mtb* and trafficking of only a subpopulation of these pathogens is influenced by the lysosomal system. In future, it will be important to understand the determinant(s) of the heterogeneous lysosomal targeting of these pathogen and its consequence for the bacteria. Such studies could lead to development of better host lysosomal targeted therapeutics against these dreaded infectious diseases.

## Author Contributions

KS and VS conceived, wrote, and edited the review. All authors contributed to the article and approved the submitted version.

## Funding

VS is supported by core funding from NCBS-TIFR.

## Conflict of Interest

The authors declare that the research was conducted in the absence of any commercial or financial relationships that could be construed as a potential conflict of interest.
